# A Conversation with ASTMH Past President Regina Rabinovich

**DOI:** 10.4269/ajtmh.18-interview

**Published:** 2019-01

## Abstract

At the 2018 ASTMH Annual Meeting, October 28 through November 1, N. Regina Rabinovich, MD, FASTMH, wrapped up her term as ASTMH President. She recently talked with science writer Matthew Davis about the highlights of her year, the current state of the fight against major threats, including malaria and Ebola, and her advice for the new Society president, Chandy C. John, MD, MS, FASTMH.

## Tell us a little bit about your past year as president. What stood out for you as particularly rewarding?

The most rewarding work involved creating the ASTMH Respect/Inclusion Policy task force. I think the statement the members drafted articulating the ASTMH Respect/Inclusion Policy is important for an organization that just hosted an Annual Meeting that included almost 5,000 people from 119 countries.

Our diversity draws from multiple cultures, languages, and ethnicities. The task force carefully considered what dignity, mutual respect, and inclusion mean for our members and how that informs our interactions. It’s critical that we articulate a clear policy and also that we are intentional in carrying it out, particularly when we see a lack of respect and inclusion.

I also appreciated the opportunity to appoint the (ASTMH Annual Meeting) keynote and to help choose a voice that represents this point in time. I think it was very fortunate that we ended up with Dr. Matshidiso Moeti (WHO Africa Regional Director). We asked her to pick a topic she was passionate about, and I thought it was very compelling and serendipitous for her to focus on the role of women in transforming what is happening in global health.

Most recently, I have been involved in efforts to explore potential strategic alliances that can extend our reach. For example, how can we work with the Multilateral Initiative on Malaria to not only support their meeting but also to ensure their voice is part of what ASTMH is doing? I also think we should be looking not only for scientific partners but also for opportunities to share our voice and our advocacy platform.

## Your comment, “There will be epidemics …” as a forecast for 2018 was used as the key element in the ASTMH Annual Meeting logo this year. It complemented an overall theme recognizing the 100^th^ anniversary of the Spanish flu and focusing on the threat of disease outbreaks. How has global preparedness for outbreaks improved in the last 3 or 4 years, particularly since the Ebola outbreak in West Africa?

I think, for Ebola specifically, we are much better prepared. There are new drug and vaccine candidates that appear to be very promising. There is a sound plan in place for how to go in and contain an outbreak, although security concerns can make that challenging to implement.

In terms of preparedness for a disease that could cause a global outbreak, my biggest concern today is around influenza because I don’t think we are prepared at all. Surveillance has improved, but we don’t have the vaccine supply and manufacturing capacity available to deal with a global pandemic.

I was at least pleased to learn that flu vaccinations were available at the Annual Meeting. Because, if there is a year when a flu season starts early, the Annual Meeting is the kind of gathering where influenza infections could spread fast.

## You have done a lot of work with malaria and malaria remains a priority for many ASTMH members. But there are some who fear that, after years of progress, the fight against malaria is stalling and could even regress. Do you share that view?

The issue with malaria is that if you let the pressure off and don’t pay sufficient attention, it can rebound really fast. Look at what’s happening with malaria in Venezuela or Yemen. There are now hundreds of thousands of infections in these countries, including in areas where malaria was close to being eliminated. But I remain optimistic about the global fight. We just have to be mindful that this is a long-term effort and, in some ways, the fastest progress involves tackling disease in areas of high transmission. Now we have to come up with strategies for fighting malaria in areas where transmission is at a lower level and where we’re dealing with a lot of asymptomatic infections. Progress in these settings will be slower and will require new tools.

**Figure:**
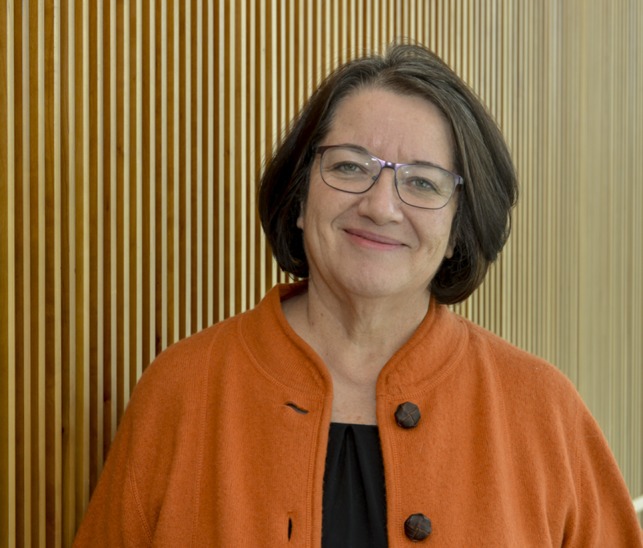
**2018 President N. Regina Rabinovich, MD, FASTMH**

Sometimes we get criticized as a field because there is a sense that we are not moving rapidly enough to eliminate a threat or that work has stalled. For example, we see criticism around the fight to eradicate diseases like polio and guinea worm because we’re not there yet. But when you get to the tail end, the work can be hard and painful. We need to help policy makers understand that if we don’t persevere and finish the job, all of the incredible amount of work that has been invested thus far will be lost.

The basic issue is that when a disease is not at an epidemic level, funding tends to dry up. Malaria is an example of a disease that can come back very quickly, but it can happen with other diseases as well.

## There was a lot of discussion at the Annual Meeting about the difficulty today of fighting infectious disease outbreaks in places like the Democratic Republic of Congo and Yemen, where the security situation is very challenging. Are we entering a time where security and logistics are becoming more challenging than the biology of the disease?

Global health work will always involve fragile states and deal with the challenges of fighting disease in very unstable environments. We’re seeing it today in places like Venezuela, the Democratic Republic of Congo, Sudan, and Yemen. But this is not something new.

The fight to eradicate smallpox and the incredible progress against polio involved working in very volatile locations. There were polio vaccination days in Northern Nigeria during times of incredible political and religious strife. More recently, in the midst of the Ebola outbreak in West Africa, there was a successful effort in Sierra Leone to distribute three million treatments for malaria.

I’m not minimizing the challenge. It’s very difficult work and the risks are real. But it’s possible to make progress if people care enough to provide the support.

## What advice do you have for Chandy John, MD, MS, FASTMH, as he embarks on his term as ASTMH President?

Part of being ASTMH President involves getting out there and being the face of the Society. It can be a demanding responsibility, but it’s also an honor. I would encourage Chandy to embrace that role and enjoy the opportunities to engage.

There will be a critical need for advocacy because, politically, it’s still a very difficult time for the issues we are passionate about, like funding for global health research and supporting evidence-based policies. We can’t cover everything and he will need to pick his priorities and consider where the Society can have the biggest impact.

But we have seen that advocacy and engagement can make a difference. There were significant achievements over the last year, like the move by the FDA to expand the list of neglected tropical diseases and the agency’s decision to approve a new drug for treating *Plasmodium vivax* malaria. But there will be no easy wins, and new, unexpected challenges can emerge at any time.

The bottom line is that, in the current environment, ASTMH leaders have to be on their game and always prepared to fight for our priorities.

